# Body Posture Stability in Ski Boots Under Conditions of Unstable Supporting Surface

**DOI:** 10.2478/hukin-2013-0043

**Published:** 2013-10-08

**Authors:** Dariusz Tchórzewski, Przemysław Bujas, Agnieszka Jankowicz-Szymańska

**Affiliations:** 1 Department of Physical Education and Sport. University School of Physical Education in Cracow, Poland.; 2 Institute of Health. State Higher Vocational School in Tarnow, Poland

**Keywords:** dynamic balance, compliant surface, ski boots, beginner skiers

## Abstract

The authors attempted to determine whether: (1) there are differences in stability between the conditions of standing in ski boots and barefoot, (2) the type of surface affects stability, and, (3) the level of stability differs between the frontal and sagittal planes. The study included 35 young male recreational skiers aged 20.71 ±0.63 years. Measurements of stability were taken by means of a Libra seesaw balance board. The conditions of soft surface were created by attaching an inflated cushion to the board. The experiment was carried out on both rigid and soft surface for both movement planes and two different conditions: maintaining the seesaw balance board in the horizontal position and performance of a particular balancing task. All the tests were performed with visual feedback. Restricted ankle joint mobility that results from wearing ski boots caused a reduction of stability in studied subjects, particularly in the sagittal plane. The differences found in the study were likely to be caused by the difficulty the beginners experienced in re-organizing muscular coordination in hip joint strategy and effectively using mechanical support of ski boots that reduces lower limb muscle tone. The use of the soft surface improved stability exhibited by the subjects in the frontal plane without compromising the stability in the sagittal plane. The soft surface might have contributed to a reduction in excessive corrective movements, thus improving stability in studied subjects. The aim of this study was to determine the effect of limitation of foot mobility and disturbances in afferent information from the plantar mechanoreceptors due to wearing ski boots on the level of postural stability in beginner skiers under conditions of the unstable support surface.

## Introduction

Maintaining the standing posture occurs in human body based on sensory information from the organ of vision, the vestibular system and the somatosensory system. These three sources of sensory information must be integrated in order to provide complex sensory interpretation of the conditions a human experiences at a particular moment (Mergner et al., 2003; Peterka and Loughlin, 2004; Erkmen et al., 2010). Their effect on balance varies considerably (Riemann and Lephart, 2002). When standing on a firm support surface, healthy young people rely in 70% on somatosensory information and in 20% on the vestibular one, with remaining 10% being visual information (Peterka, 2002). Consequently, when standing on stable ground, the major source of afferent signals used in the process of balance control is proprioceptive information obtained from muscular, articular and sensory receptors and those in constant contact with support surface of the plantar mechanoreceptors (Shumway–Cook and Horak, 1986). Under conditions of the compliant surface, the proprioceptive information is much restricted, which requires from the balance control system a fast and optimum re-organization of the degree of using afferent information from other sensory inputs (Dietz et al., 1980; Ivanenko et al., 1997; Mergner, 2010).

The opportunity for compensating for the loss or limitation of information from one or even two sources through re-organization of the importance of the sensory inputs and rearrangement of the weights of information from all the senses is important for maintaining stability when a person replaces one sensory context with another one (Mergner et al., 2003; Peterka and Loughlin, 2004; Horak, 2006). When changing the support surface from a firm into an unstable one, the importance of sensory information from the vestibular system and the organ of vision increases rapidly (Peterka, 2002).

When balance is challenged on a compliant surface while standing in normal conditions maintaining postural stability is of key importance. This is true for both everyday human activity and athletic performance. In Alpine skiing, the ability of controlling body posture is additionally limited by ski boots that restrict mobility of the ankle joint. Apart from making ankle joint more rigid, the design of a ski boot, with its cuff inclined forward, forces the change in position from natural to the inclined one (Schaff and Hauser, 1987; Bennell and Goldie, 1994). However, ski boots provide the mechanical support for the shank and are a source of additional sensory information from the sensory cutaneous receptors, which, under conditions of a firm surface, leads to the improvement in postural control (Rogers et al., 2001).

Furthermore, wearing ski boots and skis expands the support surface, which likely facilitates maintaining a vertical position. In Alpine skiing, the lower limb, in conjunction with visual perception, plays a fundamental role in transferring stimuli from the external environment to the balance control system. Unlike standing barefoot, wearing ski boots impairs the ability of sensing the ground pressure forces, which is one of the main sources of information about proper performance of a movement task (Noe and Paillard, 2005). It is necessary for foot mechanoreceptors in a ski boot to sense the ground shape through a rigid sole and a ski so that ski control is maintained. It can also be relatively more or less impaired by the type of snow on which the skier has to move. The surface of a ski slope might vary from a hard icy one to very soft, covered with freshly fallen snow (Zatoń et al., 2008).

The studies that have attempted to provide new insight into multisensory control of standing position in ski boots in both stable and unstable conditions of maintaining balance are scarce and have been carried out mainly among groups of professional athletes (Schaff and Hauser, 1987; Noe and Paillard, 2005; Noe et al., 2009). The studies carried out under stable ground conditions have demonstrated a higher level of stability when wearing ski boots compared to standing barefoot. Under conditions of an unstable surface, the differences between both variants of standing have been shown to be insignificant (Noe and Paillard, 2005).

The aim of the present study was to determine these relationships in skiers who have not yet developed specific patterns of muscular coordination typical of professional skiers used when maintaining postural stability under conditions of limitation caused by ski boots. The study was carried out using a seesaw balance board on two types of surface (firm and compliant) and in two options, with a subject maintaining the balance board in a horizontal position or performing a balancing task which consisted in purposeful inclining the board according to a pre-set pattern. Each time the subjects used visual feedback to correct the position. The authors attempted to determine whether: (1) there are differences in stability between the conditions of standing in ski boots and barefoot, (2) the type of surface affects stability, and, (3) the level of stability differs between the frontal and sagittal planes.

## Material and Methods

### Participants

The study included 35 healthy young and physically fit men aged 20.71 ±0.63 years, body height 180.26 ±5.64 cm, body mass 73.90 ±5.65 kg, BMI 22.73 ±1.41. The basic criterion for inclusion in the study group was that the subjects were beginner skiers. This was aimed at eliminating the effect of the acquired balancing skills specific to Alpine skiers. None of the subjects studied had suffered from balance disorders nor had they had injuries that might have affected the results of balance measurements. All the subjects participated in the tests voluntarily and were informed about the possibility of withdrawing from the research.

### Testing Apparatus

When measuring the stability level, the authors used a Libra seesaw balancing board manufactured by EasyTech (length: 43 cm; width: 42 cm; height: 65 cm). The testing stand was comprised of two components: a balance board with USB interface, controlled by EasyTech 2.2-001-2.0 computer software developed by the device’s manufacturer, and a computer set. The stabilometer allowed for measurements in the frontal plane (FP) and sagittal plane (SP) within the range of angular inclination of ±15°, with a maximum measurement error of 0.1°. Electrical signals obtained from a potentiometer in the measurement circuit were converted by means of an analog-to-digital converter card.

The two types of motion patterns were: straight line and the sinusoid with the amplitude of 5° and frequency of 10 cycles/min. The curvature of the balance board was set at 40 cm and 6th level (angular deviation from the ideal line: ±5°). The scope of difficulties was presented on the screen as two parallel lines distributed at both sides of a movement pattern. These parameters were determined based on the previous studies carried out on a Libra seesaw balance board (Tchórzewski et al., 2010).

**Figure 1:**
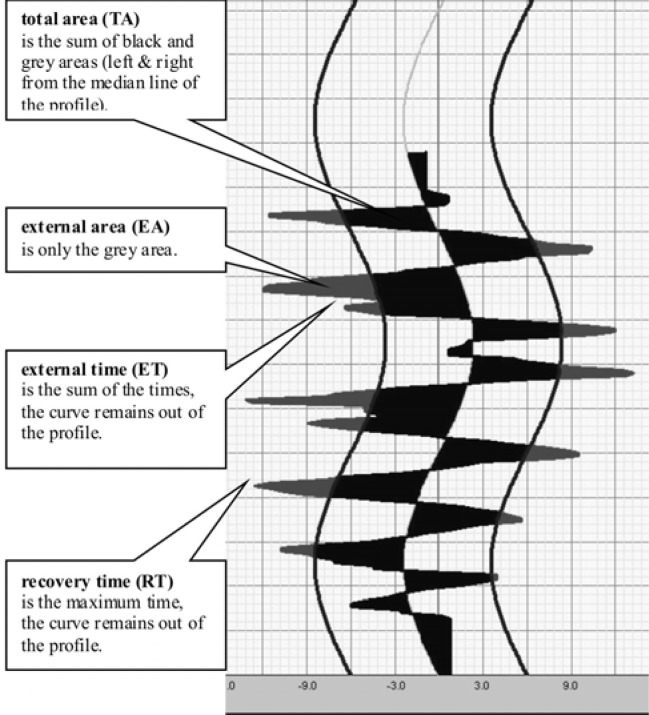
Graphic interpretation of individual stability parameters obtained on the basis of measurements made on the Libra balance platform

EasyTech 2.2-001-2.0. software converts the data obtained from the seesaw balance board that determine angular changes of position of its surface vs. time and computes four parameters of stability, separately for lateral sway in FP and anterior-posterior sway in SP (Figure 1):

○ Total Area (TA) - the area contained between the line of a movement pathway recorded for a subject and the model line. This variable is the main determinant of the level of stability, regardless of the pre-set degree of test difficulty. Its value is recomputed as a time integral of the function of the board deflection (°) from the horizontal line.
○ External Area (EA) - the area contained between the line of a movement path recorded for a subject and the line of a pre-set level of difficulty.
○ External Time (ET) - total time when a subject remains outside the area of a pre-set level of difficulty.
○ Recovery Time (RT) - the longest individual time when the subject remained outside the area of a pre-set level of difficulty.

Based on the weighted average of all the variables, the software computes the stability index (SI) within the range of 0 to 100, where 100 denotes the weakest and 0 the best stability.

In order to create the conditions of the compliant (soft) surface, a Togu Dynair cushion was used (inflated rubber cushion with pressure adjustment separately for both feet (length: 2x22 cm, width: 38 cm, height: 7 cm, pressure: 80 kPa).

### Testing Procedure

The tests were carried out for two types of standing on the balance board, separately for frontal and sagittal plane. In the first option, the subjects performed the test without ski boots (WSB), whereas in the second one, they performed in ski boots (ISB). Both options required adopting an upright relaxed positions with arms along the body, keeping feet parallel and shoulder width apart. When performing a test, the subjects were not allowed to place their palms on hips, fold their arms, put them on their thighs or to touch any part of their body in order to help the posture become more stable.

In each option, the subjects performed the test on a rigid surface (RS) or on a soft surface (SS) which was provided by an inflated cushion placed on the balance board.

The tests were carried out for different postural trials. In the 1st trial, the subject was asked to maintain the seesaw balance board in a horizontal position, whereas in the 2nd trial, they were asked to incline it according to a sinusoid pattern with the amplitude of 5° and the frequency of 10 cycles/min. This position forced a smooth transition from one inclination direction to the other one every 1.5 s.

Both trials were carried out using a visual feedback. A 15″ screen was placed at the distance of 1 m from the board's edge and at the level of eyes of a subject. It displayed a model pathway for both trials: in the form of a straight line (1st trial) or sinusoid (2nd trial) and the actual record of the attempts made by the subject. The subjects were asked in both trials to watch the screen throughout the measurement and to respond so that the line 'drawn' by them on the computer screen should suit best possible the model line (cover it).

Each of the trials was comprised of four separate measurements in FP and SP, standing without ski boots and in ski boots on a rigid surface and then on a soft surface. Measurements were carried out in the same order and based on the same procedure as determined experimentally:

- explanation of the aim of the study and presentation of the testing apparatus;
- attempt to balance in order to determine the proper feet arrangement (10–20 s);
- 30 s attempt of balancing before the proper measurement (without recording the results)
- 30 s pre-trial before the main trial;
- 60 s the main trial (recorded).

The rest periods between the measurements were on average 10 minutes, what ensured full recovery after the physical exercise connected with balancing.

### Data Analysis

The data obtained from each measurement were initially processed based on the basic descriptive statistics methods. The arithmetic means, range, standard errors of the means and standard deviations were computed. The authors used the Wilcoxon signed-rank test in order to find the differences between both related samples. Percentage values were calculated in order to determine the size of the differences. The following equations were used when comparing stability in ski boots (ISB) and without ski boots (WSB): % difference IS=[(ISISBISWSB)/ISWSB]*100; % difference TA=[(TAISBTAWSB)/TAWSB]*100, whereas the differences between standing on rigid surface (RS) and standing on soft surface (SS) were compared using: % difference IS=[(ISSS-ISRS)/ISRS]*100; % difference TA=[(TASS-TARS)/TARS]*100. Similar method was employed to calculate the relative differences in the level of stability with respect to the sagittal plane (SP) and frontal plane (FP): % difference IS=[(ISSP-ISFP)/ISFP]*100; % difference TA=[(TASP-TAFP)/TAFP]*100.

Apart from the obtained stability index (IS) calculated by the computer software, which is a general determinant of stability observed in subjects, an index of balancing precision was also adopted (IBP), which provided information about percentage fraction of the external area (EA) in total area (TA) according to the following equation: IBP=(EA/TA)*100.

The analysis was carried out by means of Statistica 10.0 software.

## Results

**Figure 2:**
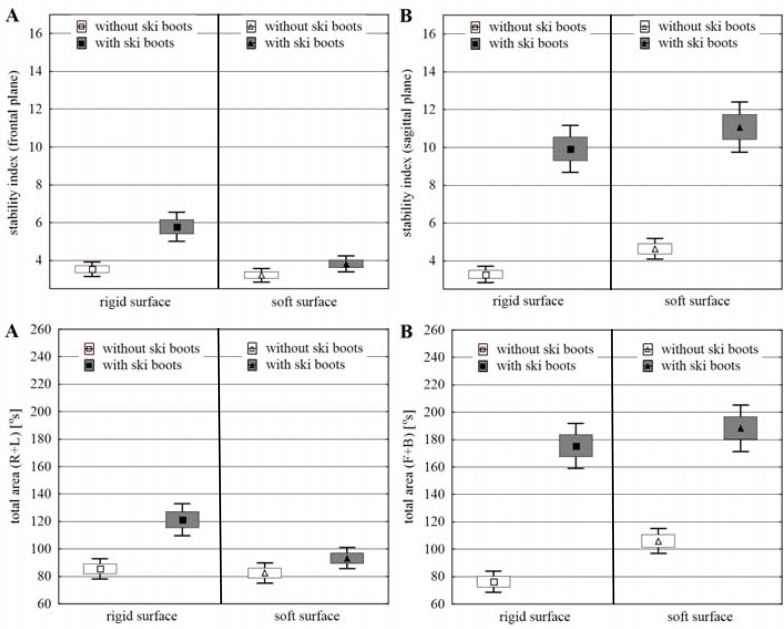
Stability index and total area under conditions of the 1st trial, considering both variants of standing in ski boots and without ski boots and the type of surface. A – frontal plane; B – sagittal plane

**Figure 3:**
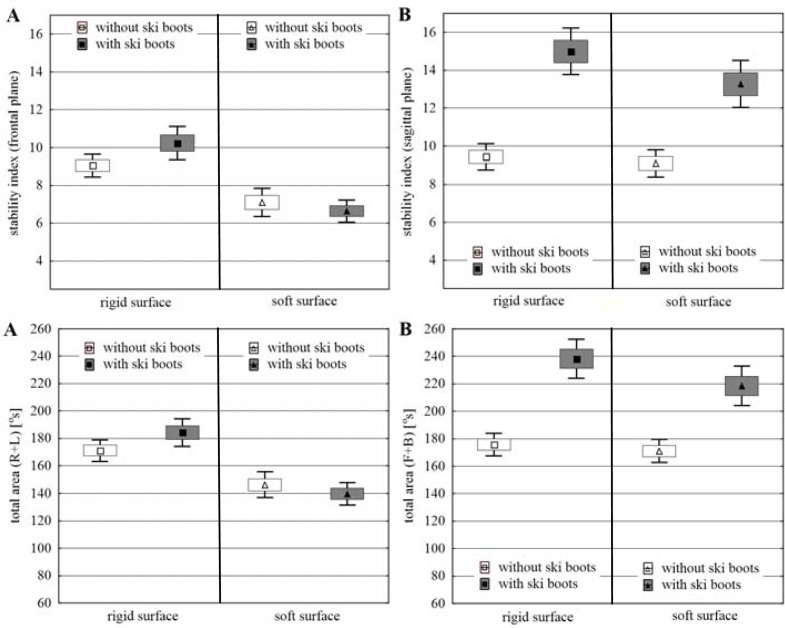
Stability index and total area under conditions of the 2nd trial, considering both variants of standing in ski boots and without ski boots and the type of surface. A – frontal plane; B – sagittal plane

The results of the measurements of stability in both options of the standing position i.e. without ski boots (WSB) and in ski boots (ISB) were considered separately for rigid surface (RS) and soft surface (SS), considering frontal (FP) and sagittal planes (SP) of movement. The obtained values of the arithmetic means, standard error of the means and 95% confidence interval of the means values of stability indices (SI) and total area (TA) in the 1st trial (maintaining the balance board in the horizontal level) were presented in Figure 2, whereas these results obtained for the 2nd trial (purposeful inclining the balance board) are shown in Figure 3.

The values for the index of balancing precision (IBP) are contained in Table 1.

### Changes in the level of stability between the conditions of standing in ski boots and without them

When balancing on the rigid surface in the 1st trial, the obtained results exhibit a higher level of stability in subjects who stood barefoot compared to those standing in ski boots, with particular focus on the sagittal plane (TA difference%: SP 130%, *p<0.000;* FP 41.8%, *p<0.000*). In case of the 2nd trial, the differences found were considerably lower (TA difference%: SP 35.5%, *p<0.000*) and statistically insignificant (Table 2).

When balancing on the soft surface, the differences between both options of standing (WSB and ISB) were considerably reduced compared to the rigid surface. In the frontal plane, they decreased for both trials and the stability level in the 2nd trial remained almost unchanged between the test in or without ski boots (TA difference%: FP 1st trial 13.1%, *p<0.002*). A reduction in differentiation in both trials was found in the sagittal plane (TA difference%: SP 1st trial 77.6%, *p<0.000;* 2nd trial 27.7%, *p<0.000*), but it still suggested a substantially higher level of stability in the subjects when standing without ski boots.

The higher precision of balancing on the rigid surface was exhibited by the subjects when standing without ski boots, particularly in the sagittal plane (IBP 1st trial FP: WSB 2.5%. ISB 6.5%; SP: WSB 3.7%. ISB 16.0%) (IBP 2nd trial FP: WSB 8.9%. ISB 12.9%; SP: WSB 10.9%. ISB 23.0%) (Table 1). The IBP levels on the soft surface exhibited similar precision for both options of standing on the balance board in the sagittal plane (IBP 1st trial FP: WSB 0.9%. ISB 1.9%; 2nd trial FP: WSB 6.2%. ISB 5.1%) and considerably higher when standing barefoot in the sagittal plane (IBP 1st trial SP: WSB 3.5%. ISB 18.7%; 2nd trial SP: WSB 10.4%. ISB 20.7%).

### Changes in the level of stability between balancing on rigid and soft surface

Differences between the parameters of stability when standing on the rigid and soft surface, both barefoot and in ski boots reveal a different effect on the level of stability in both movement planes. In the frontal plane (without ski boots), they suggest either a higher level of stability in subjects on the soft surface or the lack of this difference (TA difference%: FP 1st trial −3.6%, *p<0.611;* 2nd trial 14.5%, *p<0.000*). When balancing in ski boots, they point to a considerably higher level of stability on the soft surface (TA difference%: FP 1st trial −23.1%, *p<0,000;* 2nd trial −24.1%, *p<0.000*). Apart from the results obtained in the 1st trial (TA difference% SP: 1st trial 39.1%, *p<0.000*), the differences in the level of stability depending on the surface used were small and usually insignificant (Table 3).

When balancing without ski boots, the IBP indices (Table 1) obtained for both rigid and soft surfaces were similar, whereas these values for the trial in ski boots measured in the frontal plane turned out to be more beneficial when standing on a soft ground (IBP 1st trial: RS 6.3%. SS 1.9%; 2nd trial: RS 12.9%. SS 5.1%). This regularity was not found for the sagittal plane (IBP 1st trial: RS 16.0%. SS 18.7%; 2nd trial: RS 23.0%. SS 20.7%).

### The differences in the level of stability between the frontal and sagittal plane

In all cases and under both conditions of the rigid and soft surface, the results of stability parameters obtained by the subjects when balancing in ski boots exhibited unequivocally a higher level of stability in the frontal plane. Higher relative differences in TA in favour of the frontal plane were found when balancing on the soft (TA difference%: 1st trial 101.9%, *p<0.000;* 2nd trial 56.3%, *p<0.000*) compared to the rigid surface (TA difference%: 1st trial 44.6%, *p<0.000;* 2nd trial 29.3%, *p<0.000*) (Table 4).

When balancing without ski boots on the rigid surface, no significant differences were found between stability parameters obtained in both planes and both in the 1st and 2nd trial. The results obtained on the soft surface suggested a higher level of stability of the subjects in the frontal plane (TA difference%: 1st trial 28.6%, *p<0.000;* 2nd trial 16.9%, *p<0.000*).

The IBP indices for both planes indicate similar precision of balancing without ski boots on the rigid surface and insignificantly higher on the soft surface in the frontal plane (Table 1). When wearing ski boots, both on the soft (IBP 1st trial: FP 1.9%. SP 18.7%; 2nd trial: FP 5.1%. SP 20.7%) and rigid surface (IBP 1st trial: FP 6.3%. SP 16.0%; 2nd trial: FP 12.9%. SP 23.0%), remarkably higher precision of balancing was observed for the subjects in the frontal plane.

## Discussion

Alpine skiing is a sport where a support surface is limited and the ground is usually unstable and slippery. Feet are a body part which is critical to this sport. They are responsible for receiving sensory stimuli from the ground through the skis and boots and for precise distribution of pressure on the equipment. In beginner skiers, this process is disturbed. Therefore, from the viewpoint of coaching skiing, it is essential to determine the effect of considerable limitation in ankle joint mobility caused by wearing ski boots on the level of postural stability of a skier. It is extremely important for Alpine skiing technique that stability should be maintained in both movement planes. In the frontal plane, depending on the turning radius, a skier inclines to the right or left in order to place his/her skis on the edges. In the sagittal plane, the skier is forced to continuously control body arrangement with respect to the varying angle of the slope. Testing the stability level under conditions which are most similar to maintaining balance on skis can be carried out on a seesaw balance board.

Morphology of the cutaneous mechanoreceptors in the human foot has not been fully researched yet. The pattern of detecting dynamic changes in body posture through the cutaneous plantar mechanoreceptors also remains unknown. It is supposed that differentiation of postural movements through foot mechanoreceptors can be achieved as a result of sensing total changes in pressure in the area of metatarsus, forefoot or calcaneus or by sensing the difference between the pressure in different areas of the foot (Wu and Chiang, 1996; Chiang and Wu, 1997).

The present study, based on the measurements taken on a balance board, attempted to determine the effect of limitation of foot mobility caused by ski boots on the degree of postural stability of beginner skiers. Furthermore, the conditions of the trials were varied through different hardness of the board's surface. The experiments were carried out in both planes (FP and SP) with subjects maintaining the balance board horizontally (1st trial) and purposefully inclining the board following a pre-set pathway (2nd trial).

The results obtained in the study demonstrated that when balancing on the rigid surface of the balance board, the mechanical limitation of foot mobility caused a reduction in stability of the studied subjects. The differences with respect to standing barefoot were, however, considerably lower in the frontal compared to sagittal plane. The differences found in the study might have been caused by a variety of mechanisms of controlling balance in both planes. In the frontal plane, this consists mainly of unloading/loading the lower limbs as a result of the activity of the abductor/adductor muscles in the hip joint, where rigidity of the ankle joint is not that critical. In the sagittal plane, the rigidity of this joint makes it impossible to use the strategy of the ankle joint, forcing the strategy of the hip joint, which in studied subjects contributed to higher angular inclinations of the seesaw balance board. This considerably differentiates beginner Alpine skiers from professional athletes, who, through improvement in skiing technique, are able to use the mechanical support of the calves in the ski boots (reducing lower limb muscle tone and re-organizing muscular coordination) so that they obtain better results for stability in the sagittal plane. In a similar study, carried out among professional alpine skiers, Noe et al. (2009) found no negative effect of ski boots on the level of stability in the frontal plane, whereas the results obtained by the athletes in the sagittal plane suggested higher stability in ski boots. When standing on a rigid surface, these authors found unequivocal improvement in stability of the subjects after wearing ski boots (Noe and Paillard, 2005; Noe et al., 2009). Comparison of these results with the ones obtained in the present study suggests that sport experience of the athletes compared to beginner skiers allowed skiers to develop particular postural strategies that compensate for the limitations in feet mobility in ski boots.

Although the improvement in stability in both planes when standing in ski boots on a rigid surface seems to be justified by increasing support surface, it could be expected that the unstable surface of the seesaw balancing board might deteriorate stability compared to standing barefoot, as it did in the present study among the people without skiing experience. Rigid surface provides sufficient support for correction activity in the ankle joint strategy. However, when maintaining balance on the seesaw balance board, using this strategy in the sagittal plane is difficult and usually based on the strategy of the hip joint (Almeida et al., 2006). In the frontal plane and standing without ski boots, inclining of the seesaw balance board to the right or left causes that the feet placed on the board perform the opposed pronation and supination movements (forced by the inclination angle of the balance board). These movements occur only in the talocalcaneal articulation, which in ski boots has a considerably restricted range of motion. However, the muscular torques generated by the muscles responsible for pronation and supination are too weak to shift the projection of the centre of gravity and balance the load from both lower limbs (Winter, 1995). This necessitates activating a very strong group of hip joint abductors and adductors (Almeida et al., 2006), which are not restricted by the skiing footwear. This is likely to be responsible for considerably lower differences in the levels of stability when balancing in the frontal plane compared to the sagittal plane. This seems to be confirmed by the results obtained in the 2nd trial, where the subjects did not have difficulty changing the stiffness in the ankle joint in the frontal plane and had more problems doing so in the sagittal plane.

Introduction of a soft surface in the study was supposed to determine the importance of afferent information obtained from the mechanoreceptors in maintaining stability in ski boots when balancing on the seesaw balance board. Changing the hardness of the support surface is one of the techniques that are most frequently used in the analysis of the role of the somatosensory system in postural control (Marin et al., 1999; Patel et al., 2008). This method is based on the belief that introduction of a deformable base considerably disturbs somatosensory afferent signals which are responsible for maintaining balance, thus increasing its dependence on visual and vestibular information (Wu and Chiang, 1996).

The results of the present study suggest that the level of stability in the subjects when balancing in ski boots on the soft surface was higher than on the rigid one, whereas the change in the surface rigidity in the sagittal plane did not affect stability. This leads to the conclusion that the use of the soft surface did not deteriorate the level of stability and it even improved significantly in the frontal plane. The determination of the effect of standing on the compliant surface on the biomechanical parameters related to the articular receptors is very complex under the dynamic conditions of a seesaw balance board. The results obtained in the study by Wu and Chiang (1996) demonstrated that the soft surface did not lead to changes in angular values of rotation of the ankle joints and their rate directly after the movement of the balance board, but it caused smaller corrective movements with a particular delay. In our case, the soft surface might have contributed to a reduction in the amplitude of corrective movements, which was directly reflected by the improved stability in the subjects. This supposition seems to be confirmed by the findings of Chiang and Wu (1997), where the authors found that standing on the soft surface might affect the inputs of both articular receptors and plantar cutaneous mechanoreceptors, but it did not affect the receptors in calf muscles in initial phase of the board’s movement. In another study, these authors (Wu and Chiang, 1996) demonstrated that plantar mechanoreceptors participate in free regulation of displacements of the centre of gravity and, when limiting their afferent outputs, the information obtained from other senses is insufficient for postural control.

## Conclusions

Limitation of ankle joint mobility in studied skiers resulting from wearing ski boots caused a reduction in their stability. However, it occurred differently depending on the type of the test, movement plane and type of surface. The biggest differences were found in the 1st trial performed on the rigid ground in the sagittal plane. When performing the balancing task (2nd trial), the differences were considerably lower and they were insignificant in the frontal plane. The use of soft ground resulted in a considerable reduction in the differences in the stability level between standing in ski shoes and without them. Similar to standing on the rigid surface, the biggest differences were reported in the sagittal plane. This is likely to be caused by the different pattern of maintaining balance in both planes.

Balancing on the soft surface improved stability in the frontal plane without changes in the sagittal plane. The deformable ground might have contributed to reduction in the amplitude of corrective movements and improvement in stability of the subjects.

The results of stability parameters obtained in the subjects during balancing in ski boots, both under conditions of the rigid and soft surface, showed a higher level of stability in the frontal plane. These high differences were not found in the trials performed without ski boots. This fact suggests the essential effect of limited mobility of ankle joint on stability in the sagittal plane.

A significantly lower level of stability in the boots in the sagittal plane was observed in case of skiing students in comparison with the results of professional skiers. This raises the question of whether sensorimotor training should be included in basic ski training. This training would improve hip strategy in the sagittal plane and improve mechanical support of the leg shank in ski boots in conditions of limited ankle joint movement.

## Figures and Tables

**Table 1:** Indices of balancing precision (IBP)

		without ski boots		in ski boots	
trial	plane	rigid	soft	rigid	soft
1	frontal	2,5	0,9	6,3	1,9
	sagittal	3,7	3,5	16,0	18,7
2	frontal	8,9	6,2	12,9	5,1
	sagittal	10,9	10,4	23,0	20,7

IBP=[(TA-EA)/TA]^*^100; TA–total area; EA–external area

**Table 2:** Differences between the results obtained for the parameters of stability under conditions of balancing in ski boots and without ski boots

		rigid surface				soft surface			
trial	plane	difference (*x̄*±s_x_)	z	*p*	difference (%)	difference (*x̄*±s_x_)	z	*p*	difference (%)
		stability index							
1	frontal	2,24±0,4	4,51	**0,000**	63,4	0,60±0,2	3,30	**0,001**	18,7
	sagittal	6,65±0,7	4,92	**0,000**	202,8	6,45±0,8	4,85	**0,000**	139,3
2	frontal	1,19±0,5	2,14	**0,033**	13,1	−0,46±0,3	1,40	0,161	−6,4
	sagittal	5,54±0,7	4,82	**0,000**	58,6	4,18±0,6	4,50	**0,000**	45,9
		total area (°s)							
1	frontal	35,78±5,6	4,55	**0,000**	41,8	10,83±3,5	3,05	**0,002**	13,1
	sagittal	99,19±9,0	4,90	**0,000**	130,0	82,33±10,6	4,78	**0,000**	77,6
2	frontal	13,11±6,0	1,92	0,054	7,7	−6,54±3,4	1,66	0,096	−4,5
	sagittal	62,46±8,6	4,81	**0,000**	35,5	47,47±7,1	4,45	**0,000**	27,7

statistically essential values were distinguished in bold type

% difference IS=[(IS_ISB_-IS_WSB_)/IS_WSB_]*100; % difference TA=[(TA_ISB_-TA_WSB_)/TA_WSB_]*100

ISB–in ski boots; WSB– without ski boots; IS–stability index; TA–total area

**Table 3:** Differences between the results obtained for the parameters of stability during balancing on a rigid and soft surface

		without ski boots				in ski boots			
trial	plane	difference (*x̄*±s_x_)	z	*p*	difference (%)	difference (*x̄*±s_x_)	z	*p*	difference (%)
		stability index							
1	frontal	−0,32±0,2	1,03	0,304	−9,0	−1,96±0,3	4,65	**0,000**	−33,9
	sagittal	1,35±0,4	3,30	**0,001**	41,2	1,15±0,7	1,39	0,164	11,6
2	frontal	−1,95±0,4	3,65	**0,000**	−21,6	−3,59±0,4	4,97	**0,000**	−35,1
	sagittal	−0,36±0,6	1,08	0,280	−3,8	−1,72±0,8	2,20	**0,028**	−11,5
		total area (°s)							
1	frontal	−3,08±4,7	0,51	0,611	−3,6	−28,03±5,8	4,33	**0,000**	−23,1
	sagittal	29,81±5,8	3,81	**0,000**	39,1	12,94±8,8	1,14	0,256	7,4
2	frontal	−24,73±5,0	3,72	**0,000**	−14,5	−44,38±4,3	**5,04**	**0,000**	−24,1
	sagittal	−4,66±8,3	1,30	**0,195**	−2,6	−19,65±9,1	**2,12**	**0,034**	−8,2

statistically essential values were distinguished in bold type

% difference IS=[(IS_SS_-IS_RS_)/IS_RS_]*100; % difference TA=[(TA_SS_-TA_RS_)/TA_RS_]*100

RS–rigid surface; SS– soft surface; IS–stability index; TA–total area

**Table 4:** Differences between the results obtained for the parameters of stability in the frontal and sagittal plane

		without ski boots				in ski boots			
trial	surface	difference (*x̄*±s_x_)	z	*p*	difference (%)	difference (*x̄*±s_x_)	z	*p*	difference (%)
		stability index							
1	rigid	−0,26±0,27	0,72	0,472	−7,3	4,15±0,62	4,67	**0,000**	71,8
	soft	1,41±0,26	4,17	**0,000**	43,9	7,26±0,65	5,07	**0,000**	190,2
2	rigid	0,40±0,42	0,46	0,642	4,5	4,76±0,65	4,80	**0,000**	46,5
	soft	2,00±0,41	3,79	**0,000**	28,1	6,63±0,61	4,96	**0,000**	99,8
		total area (°s)							
1	rigid	−9,29±4,70	1,59	0,111	−10,9	54,12±8,42	4,64	**0,000**	44,6
	soft	23,59±4,45	3,91	**0,000**	28,6	95,09±8,55	5,07	**0,000**	101,9
2	rigid	4,66±5,25	0,15	0,879	2,7	54,01±7,72	4,69	**0,000**	29,3
	soft	24,73±5,03	3,82	**0,000**	16,9	78,74±6,99	4,98	**0,000**	56,3

statistically essential values were distinguished in bold type

% difference IS=[(IS_SP_-IS_FP_)/IS_FP_]*100; % difference TA=[(TA_SP_-TA_FP_)/TA_FP_]*100

SP–sagittal plane; FP–frontal plane; IS–stability index; TA–total area
